# Supplementary material for: Automated differentiation of wide QRS complex tachycardia using QRS complex polarity
Source: Commun Med (Lond). 2024 Dec 31;4:282. doi: 10.1038/s43856-024-00725-2 (PMC11688452; doi:10.1038/s43856-024-00725-2)
Supplement: Supplementary file 2 — Supplementary Information [file 43856_2024_725_MOESM2_ESM.pdf]

SUPPLEMENTAL FILE for MANUSCRIPT

Title: Automated Differentiation of Wide QRS Complex Tachycardia Using QRS Complex Polarity

Authors:

Adam M. May, MD[a]
Bhavesh B. Katbamna, MD[b]
Preet A. Shaikh, MD[a]
Sarah LoCoco, MD[a]
Elena Deych, MS[c]
Ruiwen Zhou, PhD[c]
Lei Liu, PhD[c]
Krasimira M. Mikhova, MD[a]
Rugheed Ghadban, MD[a]
Phillip S. Cuculich, MD[a]
Daniel H. Cooper, MD[a]
Thomas M. Maddox, MD, MSc[a]
Peter A. Noseworthy, MD[d]
Anthony Kashou, MD[d]

Author Affiliation:
[a] Department of Medicine, Division of Cardiovascular Diseases, Washington University School of Medicine in St. Louis
[b] Division of Cardiovascular Diseases, Loyola University Chicago, Stritch School of Medicine, Maywood, Illinois
[c] Division of Biostatistics, Washington University School of Medicine in St. Louis
[d] Department of Cardiovascular Medicine, Mayo Clinic, Rochester, Minnesota

Supplemental Figures: 6
Supplemental Tables: 16

Address for Correspondence:

Adam M. May, MD
660 S. Euclid Ave, CB 8086
St. Louis, MO 63110
217-778-0132
may.adam@wustl.edu

SUPPLEMENTAL TABLES

Supplemental Table S1: Characteristics of the Training cohort – Institution #1[1]

Training cohort	SWCT (n =242)	VT (n =179)	p-value
Diagnosing provider			
Cardiologist	84 (34.7)	16 (8.9)	2.2e-16
Heart rhythm cardiologist	92 (38.0)	155 (86.6)	
Non-cardiologist	66 (27.3)	8 (4.5)	
Clinical Characteristics			
Patient Age	70 (15)	66 (14)	0.006
Coronary artery disease	116 (47.9)	121 (67.6)	8.786e-05
Prior myocardial infarction	63 (26.0)	102 (57.0)	2.452e-10
Prior cardiac surgery	92 (38.0)	75 (41.9)	0.481
Congenital heart disease	16 (6.6)	12 (6.7)	1.0
Anti-arrhythmic drug use	36 (14.9)	97 (54.2)	p-value < 2.2e-16
Ischemic cardiomyopathy	38 (15.7)	89 (49.7)	p-value < 2.2e-16
Non-ischemic cardiomyopathy	53 (21.9)	54 (30.2)	0.07
ICD	17 (7.0)	112 (62.6)	p-value < 2.2e-16
Pacemaker	18 (7.4)	4 (2.2)	0.032
Left Ventricular Ejection Fraction (%)			
LVEF (>= 50)	140 (57.9)	48 (26.8)	8.977e-12
LVEF (49 - 31)	48 (19.8)	60 (33.5)	
LVEF (<= 30)	42 (17.4)	70 (39.1)	
LVEF Unknown	12 (5.0)	1 (0.6)	
Baseline ECG			
Ventricular pacing	15 (6.2)	67 (37.4)	3.405e-15
Bundle Branch Block	152 (62.8)	26 (14.5)	p-value < 2.2e-16
Gold Standard Diagnosis			
Yes	63 (26.0)	129 (72.1)	p-value < 2.2e-16

[1] Numbers in parentheses are percent (%) of *n* or standard deviation. Patients having a gold standard diagnosis were those with a corroborating EP or implantable intracardiac device recordings. Abbreviations: ECG, electrocardiogram; ICD, implantable cardioverter-defibrillator; LVEF, left ventricular ejection fraction; SWCT, supraventricular tachycardia; VT, ventricular tachycardia.

Supplemental Table S2: Characteristics of the Testing cohort – Institution #2[2]

Testing cohort	SWCT (n = 158)	VT (n =77)	p-value
Diagnosing provider			
Cardiologist	77 (48.7)	29 (37.7)	0.001
Heart rhythm cardiologist	36 (22.8)	35 (45.5)	
Non-cardiologist	45 (28.5)	13 (16.9)	
Clinical Characteristics			
Patient Age	65 (15)	64 (14)	0.705
Coronary artery disease	83 (52.5)	44 (57.1)	0.599
Prior myocardial infarction	71 (44.9)	43 (55.8)	0.152
Prior cardiac surgery	33 (20.9)	26 (33.8)	0.048
Congenital heart disease	8 (5.1)	2 (2.6)	0.593
Anti-arrhythmic drug use	45 (28.7)	46 (59.7)	1.937e-06
Ischemic cardiomyopathy	55 (34.8)	41 (53.2)	0.011
Non-ischemic cardiomyopathy	54 (34.2)	29 (37.7)	0.704
ICD	37 (23.4)	47 (61.0)	3.729e-08
Pacemaker	7 (4.4)	0 (0.0)	0.143
Left Ventricular Ejection Fraction (%)			
LVEF (>= 50)	68 (43.0)	10 (13.0)	9.455e-07
LVEF (49 - 31)	38 (24.1)	18 (23.4)	
LVEF (<= 30)	47 (29.7)	48 (62.3)	
LVEF Unknown	5 (3.2)	1 (1.3)	
Baseline ECG			
Ventricular pacing	11 (7.0)	23 (29.9)	7.19e-06
Bundle Branch Block	94 (59.5)	17 (22.1)	1.494e-07
Gold Standard Diagnosis			
Yes	55 (34.2)	48 (64.9)	2.705e-06

[2] Numbers in parentheses are percent (%) of *n* or standard deviation. Patients having a gold standard diagnosis were those with a corroborating EP or implantable intracardiac device recordings. Abbreviations: ECG, electrocardiogram; ICD, implantable cardioverter-defibrillator; LVEF, left ventricular ejection fraction; SWCT, supraventricular tachycardia; VT, ventricular tachycardia.

Supplemental Table S3: WCT-Polarity Codes[3]

	SWCT (n = 158)	VT (n = 77)	p-value
WCT-PC aVF			0.265
Negative	81 (51.3)	39 (50.6)	
Positive	54 (34.2)	32 (41.6)	
Equiphasic	23 (14.6)	6 (7.8)	
WCT-PC aVL			0.01
Negative	42 (26.6)	35 (45.5)	
Positive	90 (57.0)	36 (46.8)	
Equiphasic	26 (16.5)	6 (7.8)	
WCT-PC aVR			0.045
Negative	58 (36.7)	23 (29.9)	
Positive	62 (39.2)	43 (55.8)	
Equiphasic	38 (24.1)	11 (14.3)	
WCT-PC I			0.044
Negative	55 (34.8)	40 (51.9)	
Positive	85 (53.8)	31 (40.3)	
Equiphasic	18 (11.4)	6 (7.8)	
WCT-PC II			0.817
Negative	80 (50.6)	41 (53.2)	
Positive	50 (31.6)	25 (32.5)	
Equiphasic	28 (17.7)	11 (14.3)	
WCT-PC III			0.034
Negative	85 (53.8)	40 (51.9)	
Positive	49 (31.0)	33 (42.9)	
Equiphasic	24 (15.2)	4 (5.2)	
WCT-PC V1			0.044
Negative	87 (55.1)	30 (39.0)	
Positive	61 (38.6)	43 (55.8)	
Equiphasic	10 (6.3)	4 (5.2)	
WCT-PC V2			0.038
Negative	99 (62.7)	35 (45.5)	
Positive	53 (33.5)	37 (48.1)	
Equiphasic	6 (3.8)	5 (6.5)	
WCT-PC V3			0.264
Negative	100 (63.3)	46 (59.7)	
Positive	47 (29.7)	29 (37.7)	
Equiphasic	11 (7.0)	2 (2.6)	
WCT-PC V4			0.018
Negative	106 (67.1)	47 (61.0)	
Positive	33 (20.9)	27 (35.1)	
Equiphasic	19 (12.0)	3 (3.9)	
WCT-PC V5			0.045
Negative	95 (60.1)	39 (50.6)	
Positive	37 (23.4)	30 (39.0)	
Equiphasic	26 (16.5)	8 (10.4)	
WCT-PC V6			0.007
Negative	69 (43.7)	42 (54.5)	
Positive	61 (38.6)	32 (41.6)	
Equiphasic	28 (17.7)	3 (3.9)	

[3] Frequency of WCT-PC subtypes among the leads of the 12-lead ECG (Negative, Positive, and Equiphasic). Abbreviations: ECG, electrocardiogram; SWCT, supraventricular wide complex tachycardia; VT, ventricular tachycardia; WCT-PC, Wide QRS complex tachycardia- Polarity Code.

Supplemental Table S4: QRS-Polarity Shift[4]

	SWCT (n=158)	VT (n = 77)	p-value
QRS-PS –aVF			<0.001
No Shift	95 (60.1)	32 (41.6)	
Partial Shift	49 (31.0)	15 (19.5)	
Polarity Shift	14 (8.9)	30 (39.0)	
QRS-PS –aVL			<0.001
No Shift	123 (77.8)	32 (41.6)	
Partial Shift	29 (18.4)	20 (26.0)	
Polarity Shift	6 (3.8)	25 (32.5)	
QRS-PS – aVR			<0.001
No Shift	98 (62.0)	28 (36.4)	
Partial Shift	47 (29.7)	22 (28.6)	
Polarity Shift	13 (8.2)	27 (35.1)	
QRS-PS – I			<0.001
No Shift	113 (71.5)	26 (33.8)	
Partial Shift	32 (20.3)	21 (27.3)	
Polarity Shift	13 (8.2)	30 (39.0)	
QRS-PS – II			<0.001
No Shift	114 (72.2)	30 (39.0)	
Partial Shift	28 (17.7)	23 (29.9)	
Polarity Shift	16 (10.1)	24 (31.2)	
QRS-PS – III			<0.001
No Shift	114 (72.2)	32 (41.6)	
Partial Shift	31 (19.6)	18 (23.4)	
Polarity Shift	13 (8.2)	27 (35.1)	
QRS-PS – V1			<0.001
No Shift	129 (81.6)	24 (31.2)	
Partial Shift	13 (8.2)	11 (14.3)	
Polarity Shift	16 (10.1)	42 (54.5)	
QRS-PS – V2			<0.001
No Shift	125 (79.1)	32 (41.6)	
Partial Shift	15 (9.5)	10 (13.0)	
Polarity Shift	18 (11.4)	35 (45.5)	
QRS-PS – V3			<0.001
No Shift	126 (79.7)	42 (54.5)	
Partial Shift	18 (11.4)	3 (3.9)	
Polarity Shift	14 (8.9)	32 (41.6)	
QRS-PS – V4			<0.001
No Shift	120 (75.9)	45 (58.4)	
Partial Shift	22 (13.9)	8 (10.4)	
Polarity Shift	16 (10.1)	24 (31.2)	
QRS-PS – V5			<0.001
No Shift	98 (62.0)	35 (45.5)	
Partial Shift	43 (27.2)	17 (22.1)	
Polarity Shift	17 (10.8)	25 (32.5)	
QRS-PS – V6			<0.001
No Shift	95 (60.1)	32 (41.6)	
Partial Shift	49 (31.0)	15 (19.5)	
Polarity Shift	14 (8.9)	30 (39.0)	

[4] Frequency of QRS-PS subtypes among the leads of the 12-lead ECG (No shift, Partial shift, and Polarity Shift). Abbreviations: ECG, electrocardiogram; SWCT, supraventricular wide complex tachycardia; VT, ventricular tachycardia; QRS-PS, QRS Polarity Shift.

Supplemental Table S5: Part 1: Diagnostic performance ML Model Training [5]

	Accuracy	Sensitivity	Specificity	AUC
Logistic Regression	0.818	0.756	0.863	0.891
Artificial Neural Network	0.817	0.760	0.860	0.887
Random Forest	0.821	0.757	0.869	0.905
Support Vector Machine	0.831	0.779	0.869	0.911
Ensemble Learner	0.828	0.775	0.868	0.908

[5] Summary of the diagnostic performance of various ML model subtypes. Abbreviations: AUC, area under the curve; ML, machine learning.

Supplemental Table S6: Part 1: ML model comparisons[6]

Part 1	Gold Standard	Non-Gold Standard
SVM vs RF	0.296	0.556
SVM vs ANN	0.084	0.894
SVM vs LR	0.179	0.605
SVM vs EL	0.500	0.048*
RF vs LR	0.306	0.557
RF vs ANN	0.221	0.797
RF vs EL	0.792	0.839
ANN vs LR	0.572	0.245
EL vs LR	0.074	0.225
EL vs ANN	0.006*	0.625

[6] Comparison of fit with Delong's test. * denotes a significant difference ($p < 0.05$). Abbreviations: ANN, artificial neural network; EL, ensembler learner; LR, logistic regression; ML, machine learning; RF, random forest; SVM, support vector machine.

Supplemental Table S7: Tuning parameters for ML model subtypes in Study Part 1[7]

Tuning Object	Parameters	Default	Tuning range	Tuning parameters in the final model	Description
Logistic Regression with backward stepwise selection by AIC	Number of predictors	None	1-26	9	The number of predictors selected with AIC (Akaike Information Criterion)
Artificial Neural Network	Size	None	1-10	9	Size is the number of units in hidden layer.
	Decay	0	0.1-0.5	0.5	Decay is the regularization parameter to avoid over-fitting.
Random Forest	ntree	500	(500,1000,1500)	1500	Number of branches will grow after each time split.
	mtry	7	1-15	4	Number of variables is randomly collected to be sampled at each split time.
Support Vector Machine	C parameter	C=1	(0.75, 0.9, 1, 1.1, 1.25)	0.9	C parameter adds a penalty for each misclassified data point.
	Sigma	0.1	(0.01,0.015, 0.2)	0.01	Low values of sigma indicate a large similarity radius which results in more points being grouped together.
Ensemble Learner	The ensemble learning model is the collective average of probabilities from the logistic regression, artificial neural network, random forest, and support vector machine models.				

[7] Abbreviations: AIC, Akaike Information Criterion.

Supplemental Table S8: Importance scores for RF Models in Part 1[8]

Independent variables	Importance scores
WCT QRS duration	100.00
PMonoTVA	95.158
WCT-PC Lead aVL	24.055
WCT-PC Lead II	18.891
WCT-PC Lead I	17.864
WCT-PC Lead V3	17.357
WCT-PC Lead V1	13.792
WCT-PC Lead aVR	13.462
WCT-PC Lead V6	13.058
WCT-PC Lead V4	9.948
WCT-PC Lead V2	8.151
WCT-PC Lead III	5.172
WCT-PC Lead V5	2.414
WCT-PC Lead aVF	0.00

[8] Abbreviations: PMonoTVA, percent monomorphic time-voltage area; RF, random forest; WCT, wide complex tachycardia; WCT-PC, WCT polarity code.

Supplemental Table S9: Part 2: Diagnostic performance of ML Model Training [9]

	Accuracy	Sensitivity	Specificity	AUC
Logistic Regression	0.867	0.820	0.902	0.942
Artificial Neural Network	0.872	0.833	0.899	0.946
Random Forest	0.885	0.862	0.902	0.949
Support Vector Machine	0.869	0.828	0.898	0.945
Ensemble Learner	0.880	0.843	0.908	0.952

[9] Summary of the diagnostic performance of various ML model subtypes. Abbreviations: AUC, area under the curve; ML, machine learning.

Supplemental Table S10: Part 2: ML model comparisons[10]

Part 2	Gold Standard	Non-Gold Standard
SVM vs RF	0.709	0.740
SVM vs ANN	0.230	0.250
SVM vs LR	0.302	0.608
SVM vs EL	0.599	0.800
RF vs LR	0.185	0.399
RF vs ANN	0.151	0.111
RF vs EL	0.326	0.476
ANN vs LR	0.593	0.779
EL vs LR	0.148	0.375
EL vs ANN	0.110	0.093

[10] Comparison of fit with Delong's test. * denotes a significant difference ($p < 0.05$). Abbreviations: ANN, artificial neural network; EL, ensembler learner; LR, logistic regression; ML, machine learning; RF, random forest; SVM, support vector machine.

Supplemental Table S11: Tuning parameters for ML model subtypes in Study Part 2[11]

Tuning Object	Parameters	Default	Tuning range	Tuning parameters in the final model	Description
Logistic Regression with backward stepwise selection by AIC	Number of predictors	None	1-26	11	The number of predictors selected with AIC (Akaike Information Criterion)
Artificial Neural Network	Size	None	1-10	6	Size is the number of units in hidden layer.
	Decay	0	0.1-0.5	0.3	Decay is the regularization parameter to avoid over-fitting.
Random Forest	ntree	500	(500,1000,1500)	1500	Number of branches will grow after each time split.
	mtry	7	1-15	3	Number of variables is randomly collected to be sampled at each split time.
Support Vector Machine	C parameter	C=1	(0.75, 0.9, 1, 1.1, 1.25)	1.25	C parameter adds a penalty for each misclassified data point.
	Sigma	0.1	(0.01,0.015,0.2)	0.01	Low values of sigma indicate a large similarity radius which results in more points being grouped together.
Ensemble Learner	The ensemble learning model is the collective average of probabilities from the logistic regression, artificial neural network, random forest, and support vector machine models.				

[11] Abbreviations: AIC, Akaike Information Criterion.

Supplemental Table S12: Importance scores for RF Models in Part 2[12]

Independent variables	Importance scores
WCT QRS duration	100.00
PMonoTVA	69.46
QRS-PS Lead V1	68.59
QRS-PS Lead aVL	51.40
QRS-PS Lead aVF	45.59
QRS-PS Lead I	43.22
QRS-PS Lead III	41.60
QRS-PS Lead V3	38.43
QRS-PS Lead II	31.08
QRS-PS Lead aVR	30.32
QRS-PS Lead V2	28.18
QRS-PS Lead V6	23.72
QRS-PS Lead V4	22.53
QRS-PS Lead V5	0.00

[12] Abbreviations: PMonoTVA, percent monomorphic time-voltage area; QRS-PS, QRS polarity shift; RF, random forest; WCT, wide complex tachycardia.

Supplemental Table S13: Part 3: Diagnostic performance of ML Model Training [13]

	Accuracy	Sensitivity	Specificity	AUC
Logistic Regression	0.837	0.802	0.862	0.899
Artificial Neural Network	0.857	0.829	0.878	0.932
Random Forest	0.877	0.854	0.893	0.946
Support Vector Machine	0.870	0.842	0.891	0.941
Ensemble Learner	0.865	0.833	0.888	0.943

[13] Summary of the diagnostic performance of various ML model subtypes. Abbreviations: AUC, area under the curve; ML, machine learning.

Supplemental Table S14: Part 3: ML model comparisons[14]

Part 3	Gold Standard	Non-Gold Standard
SVM vs RF	0.271	0.030*
SVM vs ANN	0.001*	0.395
SVM vs LR	< 0.001*	0.009*
SVM vs EL	0.006*	0.662
RF vs LR	< 0.001*	0.001*
RF vs ANN	0.003*	0.131
RF vs EL	0.019*	0.034*
ANN vs LR	< 0.001 *	0.014*
EL vs LR	< 0.001*	0.002*
EL vs ANN	0.019*	0.301

[14] Comparison of fit with Delong's test. * denotes a significant difference ($p < 0.05$). Abbreviations: ANN, artificial neural network; EL, ensembler learner; LR, logistic regression; ML, machine learning; RF, random forest; SVM, support vector machine.

Supplemental Table S15: Tuning parameters for ML model subtypes in Study Part 3[15]

Tuning Object	Parameters	Default	Tuning range	Tuning parameters in the final model	Description
Logistic Regression with backward stepwise selection by AIC	Number of predictors	None	1-50	38	The number of predictors selected with AIC (Akaike Information Criterion)
Artificial Neural Network	Size	None	1-10	3	Size is the number of units in hidden layer.
	Decay	0	0.1-0.5	0.3	Decay is the regularization parameter to avoid over-fitting.
Random Forest	ntree	500	(500,1000,1500)	1000	Number of branches will grow after each time split.
	mtry	7	1-15	6	Number of variables is randomly collected to be sampled at each split time.
Support Vector Machine	C parameter	C=1	(0.75, 0.9, 1, 1.1, 1.25)	1.25	C parameter adds a penalty for each misclassified data point.
	Sigma	0.1	(0.01,0.015,0.2)	0.01	Low values of sigma indicate a large similarity radius which results in more points being grouped together.
Ensemble Learner	The ensemble learning model is the collective average of probabilities from the logistic regression, artificial neural network, random forest, and support vector machine models.				

[15] Abbreviations: AIC, Akaike Information Criterion.

Supplemental Table S16: Importance scores for RF Models in Part 3[16]

Independent variables	Importance scores
WCT QRS duration	100.00
PMonoTVA	95.16
QRS-PS Lead V1	81.06
QRS-PS Lead aVL	74.79
QRS-PS Lead aVF	66.11
QRS-PS Lead III	61.13
QRS-PS Lead II	58.79
QRS-PS Lead I	54.11
QRS-PS Lead V3	52.85
QRS-PS Lead V6	47.16
QRS-PS Lead V4	46.17
QRS-PS Lead aVR	45.38
QRS-PS Lead V2	44.53
QRS-PS Lead V5	32.18
WCT-PC Lead aVL	24.06
WCT-PC Lead II	18.89
WCT-PC Lead I	17.86
WCT-PC Lead V3	17.36
WCT-PC Lead V1	13.79
WCT-PC Lead aVR	13.46
WCT-PC Leads aVF, III, V2, V4, V5, and V6 (multiple)	0.00

[16] Abbreviations: PMonoTVA, percent monomorphic time-voltage area; RF, random forest; QRS-PS, QRS polarity shift; WCT, wide complex tachycardia; WCT-PC, WCT polarity code.

SUPPLEMENTAL FIGURES

Supplemental Figure S1: Three Part Study Design [17]

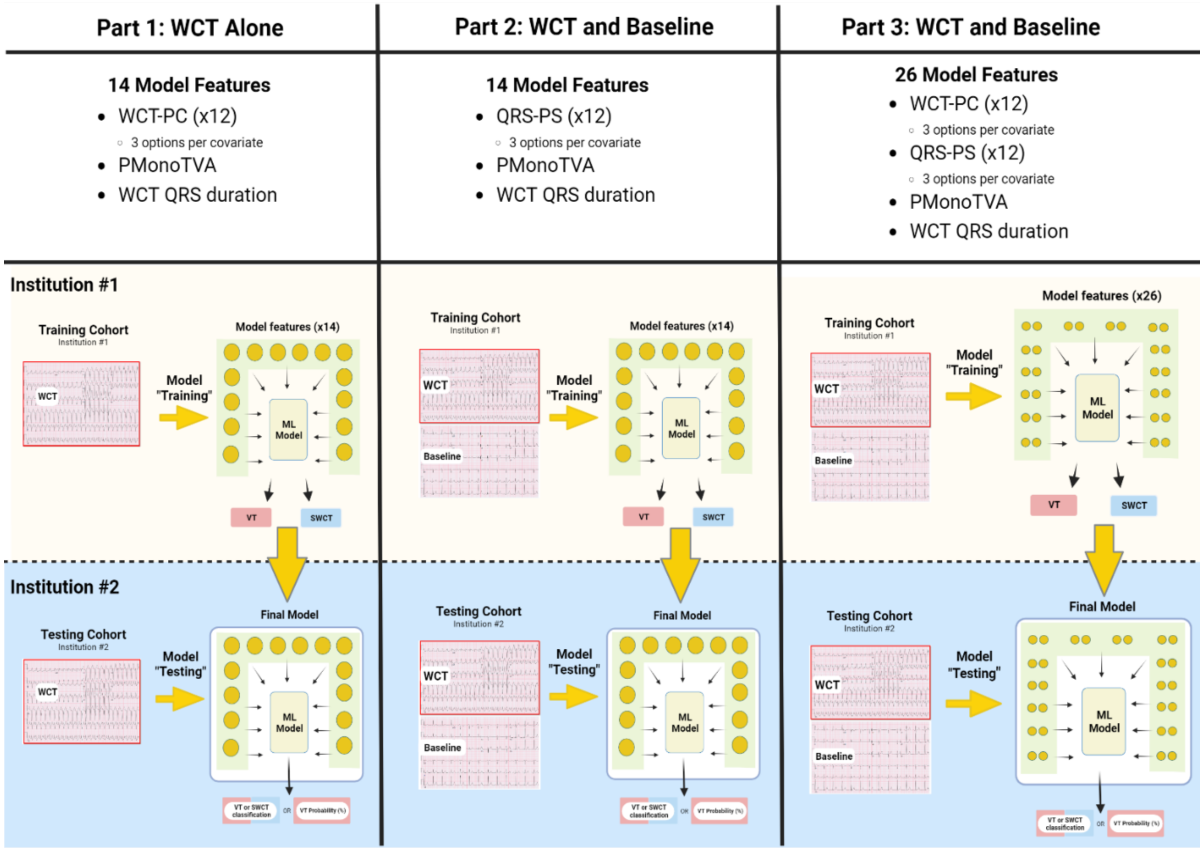

[17] Three part study design. Each part used differing set of model features to be incorporated in ML learning models. Model training used ECGs acquired at Institution #1. Model testing used ECGs acquired at Institution #2. Abbreviations: ML, machine learning; PMonoTVA, percent monomorphic time voltage area; QRS-PS, QRS Polarity Shift; QRS-PS, QRS Polarity Shift; SWCT, supraventricular wide complex tachycardia; VT, ventricular tachycardia; WCT, wide complex tachycardia; WCT-PC, WCT Polarity Code. *Created with BioRender.com.*

Supplemental Figure S2: Testing Cohort Selection[18]

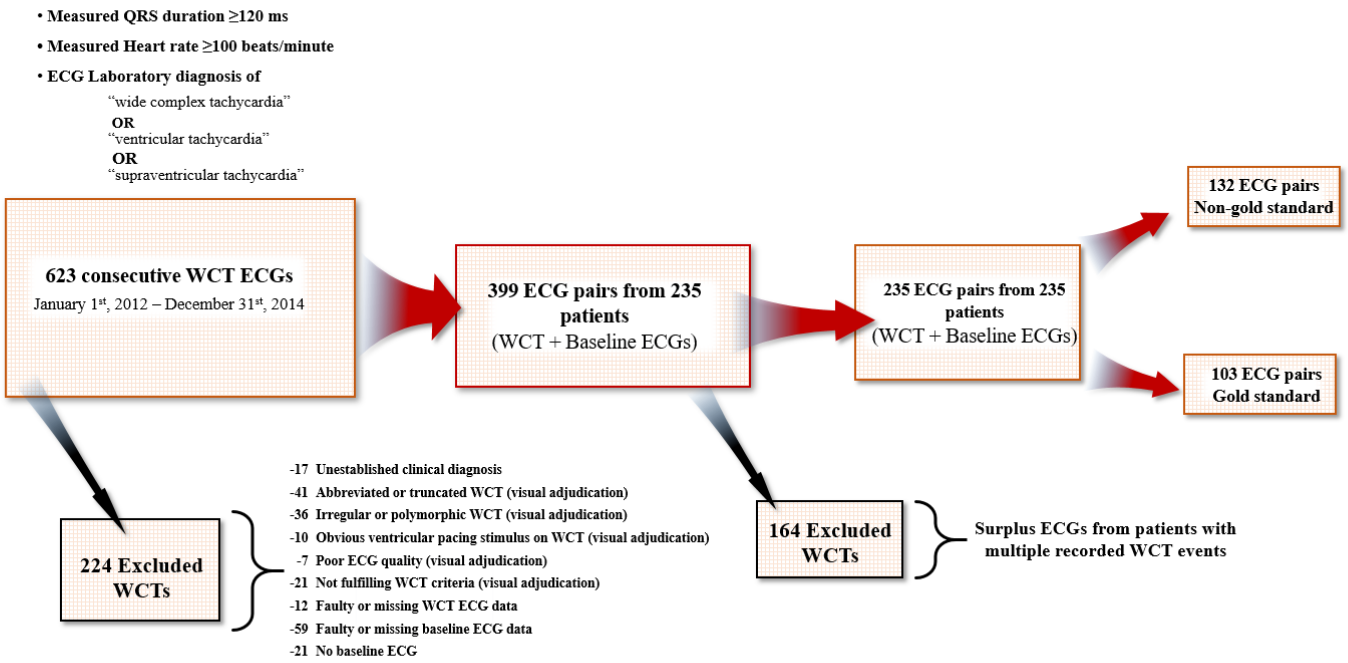

[18] Selection of WCT ECGs for the testing cohort (Institution #2). Flow diagram depicting WCT ECG cohort selection. Abbreviations: ECG, electrocardiogram; WCT, wide complex tachycardia.

Supplemental Figure S3: QRS Complex Measurements[19]

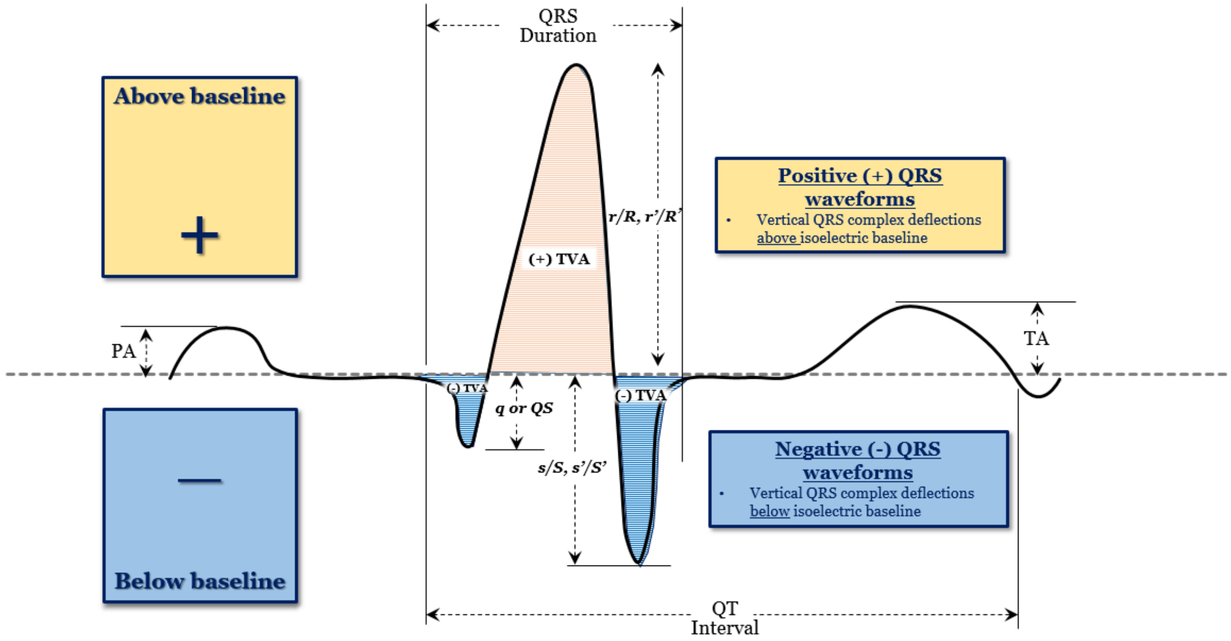

[19] Measurements of QRS complex waveforms. Schematic representation of a QRS complex and its measurable components provided by computerized ECG interpretation software. QRS amplitude (μV) represents the vertical height of positive (r/R and r'/R') and negative (q/QS, s/S, and s'/S') QRS waveforms. QRS time-voltage areas (TVAs) (μV·ms) represents the "area" enveloped by individual QRS complex waveforms above (r/R and r'/R') or below (q/QS, s/S, and s'/S') the isoelectric baseline. Abbreviations: PA, P- wave amplitude; TA, T- wave amplitude; TVA, time-voltage area.

Supplemental Figure S4: PMonoTVA[20]

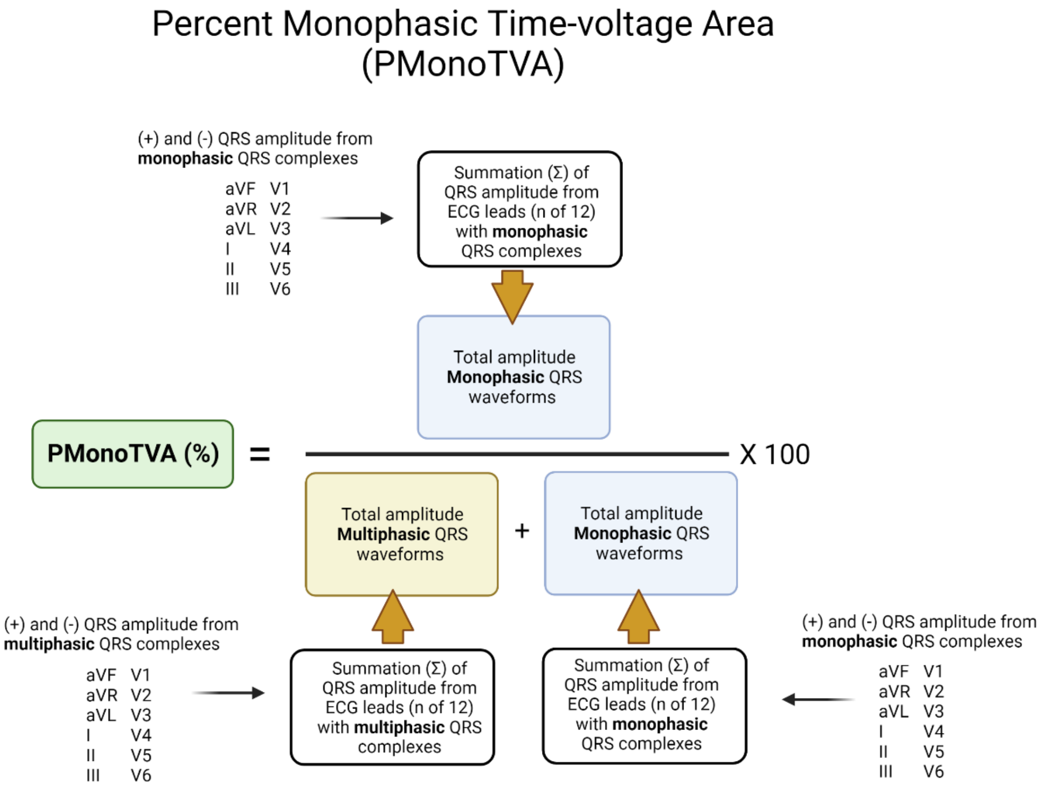

[20] Percent monophasic time-voltage area calculation. PMonoTVA calculation is derived from measured QRS waveform TVA (time-voltage areas) (µV·ms) of the dominant QRS complex template within each lead of the 12-lead ECG. Abbreviations: ECG, electrocardiogram; PMonoTVA, percent monophasic time voltage area; TVA, time voltage area; WCT, wide complex tachycardia. *Created with BioRender.com.*

Supplemental Figure S5: Examples of QRS Polarity Shift [21]

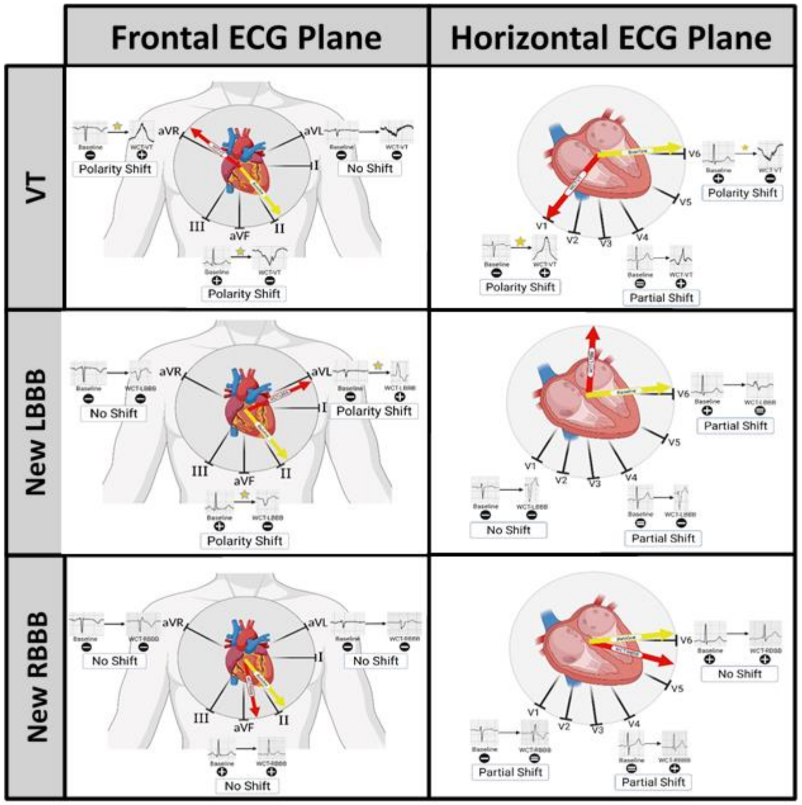

[21] Examples of QRS Polarity Shift (QRS-PS) among examples of VT and SWCT. The changes in mean electrical vector from a baseline ECG into (i) VT, (ii) SWCT due to LBBB, and (iii) SWCT due to RBBB are demonstrated in the frontal (left panels) and horizontal plane (right panels). The polarity of QRS complexes are defined as positive (+), negative (-), or equiphasic (=) based upon the directionality of the QRS complex waveform. Colored arrows represent the mean electrical vector of the WCT (red) and baseline (yellow) rhythms. Compared to new RBBB or LBBB, VT has multiple polarity shifts across the ECG leads shown. LBBB, left bundle branch block; RBBB, right bundle branch block; VT, ventricular tachycardia; WCT, wide QRS complex tachycardia. *Created with BioRender.com.*

Supplemental Figure S6: WCT Differentiation Algorithm Application[22]

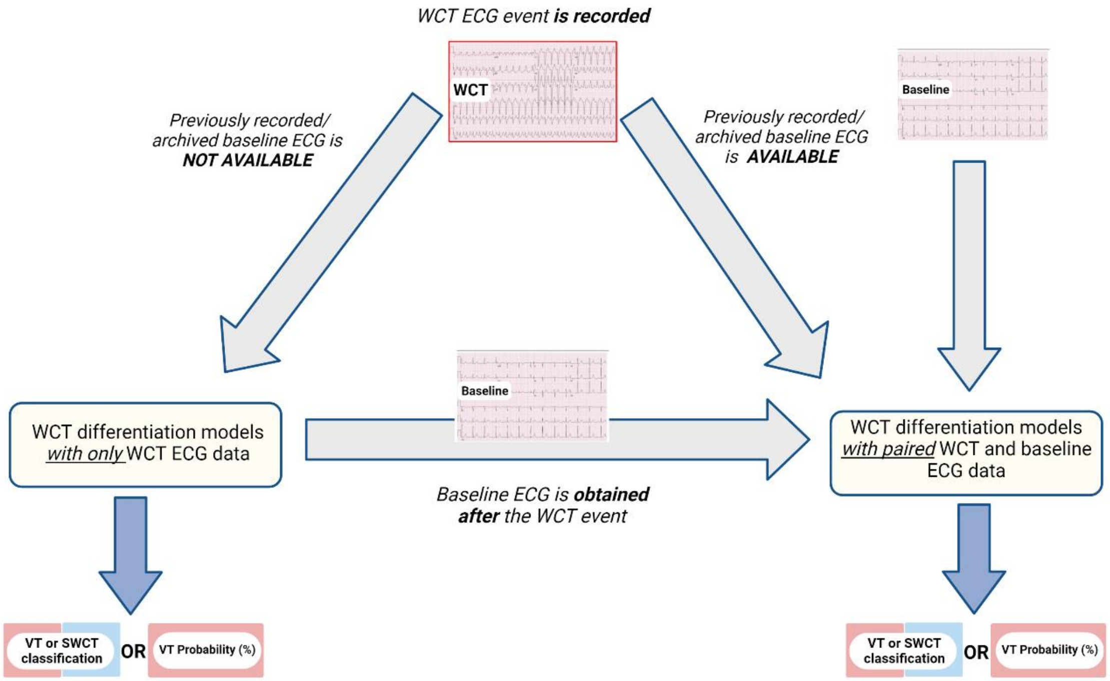

[22] Proposed process for WCT differentiation algorithm application as determined by the presence or absence of a baseline ECG. Abbreviations: ECG, electrocardiogram; SWCT, supraventricular wide complex tachycardia; VT, ventricular tachycardia; WCT, wide complex tachycardia. *Created with BioRender.com.*

127
128
129
130
131
