# Supplementary material for: Automated differentiation of wide QRS complex tachycardia using QRS complex polarity
Source: Commun Med (Lond). 2024 Dec 31;4:282. doi: 10.1038/s43856-024-00725-2 (PMC11688452; doi:10.1038/s43856-024-00725-2)
Supplement: Supplementary file 3 — Description of Additional Supplementary Files [file 43856_2024_725_MOESM3_ESM.pdf]

Description of Additional Supplementary Files

File name: Supplementary Data File D1

File description: The frequency of various types of QRS-PSs for VT and SWCT groups within the testing cohort

File name: Supplementary Data File D2

File description: Source data for Figures 3 and 4
