# Supplementary material for: Automated differentiation of wide QRS complex tachycardia using QRS complex polarity
Source: Commun Med (Lond). 2024 Dec 31;4:282. doi: 10.1038/s43856-024-00725-2 (PMC11688452; doi:10.1038/s43856-024-00725-2)
Supplement: Supplementary file 6 — Reporting Summary [file 43856_2024_725_MOESM6_ESM.pdf]

Corresponding author(s): Adam May, MD

Last updated by author(s): Nov 20, 2024

Reporting Summary

Nature Portfolio wishes to improve the reproducibility of the work that we publish. This form provides structure for consistency and transparency in reporting. For further information on Nature Portfolio policies, see our Editorial Policies and the Editorial Policy Checklist.

Statistics

For all statistical analyses, confirm that the following items are present in the figure legend, table legend, main text, or Methods section.

n/a	Confirmed	
☐	☒	The exact sample size (*n*) for each experimental group/condition, given as a discrete number and unit of measurement
☒	☐	A statement on whether measurements were taken from distinct samples or whether the same sample was measured repeatedly
☐	☒	The statistical test(s) used AND whether they are one- or two-sided<br>*Only common tests should be described solely by name; describe more complex techniques in the Methods section.*
☐	☒	A description of all covariates tested
☒	☐	A description of any assumptions or corrections, such as tests of normality and adjustment for multiple comparisons
☐	☒	A full description of the statistical parameters including central tendency (e.g. means) or other basic estimates (e.g. regression coefficient) AND variation (e.g. standard deviation) or associated estimates of uncertainty (e.g. confidence intervals)
☒	☐	For null hypothesis testing, the test statistic (e.g. *F*, *t*, *r*) with confidence intervals, effect sizes, degrees of freedom and *P* value noted<br>*Give P values as exact values whenever suitable.*
☒	☐	For Bayesian analysis, information on the choice of priors and Markov chain Monte Carlo settings
☒	☐	For hierarchical and complex designs, identification of the appropriate level for tests and full reporting of outcomes
☒	☐	Estimates of effect sizes (e.g. Cohen's *d*, Pearson's *r*), indicating how they were calculated

Our web collection on statistics for biologists contains articles on many of the points above.

Software and code

Policy information about availability of computer code

Data collection	N/A
Data analysis	All statistical analysis was performed using R Statistical Software (R Foundation for Statistical Computing, Vienna, Austria).

For manuscripts utilizing custom algorithms or software that are central to the research but not yet described in published literature, software must be made available to editors and reviewers. We strongly encourage code deposition in a community repository (e.g. GitHub). See the Nature Portfolio guidelines for submitting code & software for further information.

Data

Policy information about availability of data

All manuscripts must include a data availability statement. This statement should provide the following information, where applicable:

- Accession codes, unique identifiers, or web links for publicly available datasets
- A description of any restrictions on data availability
- For clinical datasets or third party data, please ensure that the statement adheres to our policy

The data utilized in this study comprises patients' medical records, and the datasets employed in the analysis were created, processed, and securely stored to safeguard patient privacy and anonymity. The data is not publicly disclosed to ensure the protection of patient privacy but can be made accessible upon reasonable request from other clinician scientists.

Human research participants

Policy information about studies involving human research participants and Sex and Gender in Research.

Reporting on sex and gender	Sex and gender analyses were not a central component of our study. Sex and gender assignments of individual patient subjects were sourced from the electronic medical record, which includes self-reported data from patients.
Population characteristics	Patient population characteristics are described in Study Cohorts section the of the Results. Specific characteristics of population cohorts are described in Tables 1 and 2.
Recruitment	In this retrospective analysis, study subjects were not directly recruited. Instead, inclusion and exclusion criteria for adult patients (aged 18 or older) were determined based on specific characteristics related to 12-lead acquisition, ECG measurements, ECG interpretive statements, and clinical diagnosis. Comprehensive details regarding these criteria are provided in the Methods section of the manuscript.
Ethics oversight	The Mayo Clinic Institutional Review Board and the Human Research Protection Office of Washington University in St. Louis approved the retrospective data acquisition and analysis for the study subjects who presented at their respective institutions.

Note that full information on the approval of the study protocol must also be provided in the manuscript.

Field-specific reporting

Please select the one below that is the best fit for your research. If you are not sure, read the appropriate sections before making your selection.

☒ Life sciences ☐ Behavioural & social sciences ☐ Ecological, evolutionary & environmental sciences

For a reference copy of the document with all sections, see nature.com/documents/nr-reporting-summary-flat.pdf

Life sciences study design

All studies must disclose on these points even when the disclosure is negative.

Sample size	Patient subject data was collected for analysis in a retrospective fashion. No specific calculation was used to determine the sample size of derivation and testing cohorts.
Data exclusions	We established predefined inclusion and exclusion criteria for adult patients aged 18 and above, guided by specific characteristics related to 12-lead acquisition, ECG measurements, ECG interpretive statements, and clinical diagnosis. You can find comprehensive details outlining these criteria in the Methods section of the manuscript.
Replication	N/A
Randomization	Randomization was not necessary in this retrospective analysis.
Blinding	Blinding was not necessary in this retrospective analysis.

Reporting for specific materials, systems and methods

We require information from authors about some types of materials, experimental systems and methods used in many studies. Here, indicate whether each material, system or method listed is relevant to your study. If you are not sure if a list item applies to your research, read the appropriate section before selecting a response.

Materials & experimental systems

n/a	Involved in the study	
☒	☐	Antibodies
☒	☐	Eukaryotic cell lines
☒	☐	Palaeontology and archaeology
☒	☐	Animals and other organisms
☒	☐	Clinical data
☒	☐	Dual use research of concern

Methods

n/a	Involved in the study	
☒	☐	ChIP-seq
☒	☐	Flow cytometry
☒	☐	MRI-based neuroimaging
